# The causality between gut microbiota and functional dyspepsia: A two-sample Mendelian randomization analysis

**DOI:** 10.1097/MD.0000000000040180

**Published:** 2024-10-25

**Authors:** Xiaojing Jin, Keli Xu, Jingyi Wu, Chenxi Yang, Jie Bao, Lijun Du, Binrui Chen, Xiaomei Shao, Chuanlong Zhou

**Affiliations:** a The Third Clinical Medical College of Zhejiang Chinese Medical University, Hangzhou, China; b Basic Medical School, Zhejiang Chinese Medical University, Hangzhou, China; c Sir Run Run Shaw Hospital, Zhejiang University School of Medicine, Hangzhou, China; d Department of Acupuncture and Moxibustion, The Third Affiliated Hospital of Zhejiang Chinese Medical University, Hangzhou, China; e Acupuncture and Moxibustion Teaching and Research Section, The Third Clinical Medical College of Zhejiang Chinese Medical University, Hangzhou, China.

**Keywords:** functional dyspepsia, gut microbiota, Mendelian randomization

## Abstract

To investigate the potential link between gut microbiota and functional dyspepsia (FD). Genome-wide association studies (GWAS) of gut microbiota and FD were used in Mendelian randomization (MR) research. Using the GWAS of 18,340 people, instrumental variables related to gut microbiota as an exposure factor were identified. In a GWAS investigation, 189,695 control individuals and 4376 FD patients were included as outcome variables. The primary analysis technique was inverse variance weighted analysis. The reliability of MR analysis results is tested using sensitivity analysis. Two-sample Mendelian randomization analysis revealed the presence of 7 gut microbiota associated to FD. In the inverse variance weighted analysis method, Order Erysipelotrichales (odds ratio (OR): 1.301; 95% confidence interval (CI): 1.016, 1.665; *P* = .037), Family Erysipelotrichales (OR: 1.301; 95% CI: 1.016, 1.665; *P* = .037), Genus Haemophilus (OR: 1.236; 95% CI 1.059, 1.442; *P* = .007), Genus Ruminiclostridium 9 (OR: 1.422; 95% CI: 1.078, 1.877; *P* = .013), Genus Lachnospiraceae NK4A 136 group (OR: 1.297; 95% CI: 1.059, 1.589; *P* = .012) was positively associated with FD. Class Gammaproteobacteria (OR: 0.705; 95% CI: 0.522, 0.952; *P* = .022) and Genus Erysipelatoclostridium (OR: 0.747; 95% CI: 0.628, 0.888; *P* = .001) were found to be inversely related to FD. There was no evidence of pleiotropy or heterogeneity in the sensitivity analysis. Our research provides evidence for a possible link between FD and a number of gut microbiota. The role that gut microbiota plays in the development of FD requires more investigation.

## 1. Introduction

Functional dyspepsia (FD) is a frequent gut–brain interaction condition^[1]^ characterized by postmeal fullness, early satiety, midepigastric pain or burning. According to the Rome IV standard,^[2]^ FD is classified into 2 subtypes: Epigastric Pain Syndrome and Postprandial Discomfort Syndrome. The global prevalence of FD is around 16%,^[3]^ but it varies substantially by geographic region. At the moment, Western medicine’s clinical treatment^[1,4]^ mostly employs acid suppressive preparations, gastric motivating medications, digestive enzyme preparations, probiotics, and neuromodulators to reduce chronic symptoms.^[5]^ However, this sort of treatment leads in frequent physician visits, severe physical and psychological distress, increased disease burden, and decreased quality of life.^[3,6]^ The etiology and pathophysiology of FD remain unknown. According to several studies, FD is associated with a brain–gut interaction problem involving gut microbiota disruption, visceral hypersensitivity, gastrointestinal motility abnormality, neural immune network, mental and psychological condition, and other aspects.^[3,7]^

According to Rome IV, brain–gut interaction (brain–gut axis) disturbance is a key pathogenesis of FD, impacting gastrointestinal immunity, microecology, nervous system control, and other variables.^[8]^ There is mounting evidence that the gut flora has a role in the etiology of FD. The amount of streptococcus on duodenal mucosa was larger in FD patients than in healthy participants, although the number of prevotella, Veronella, and actinomyces was lower.^[9]^ Furthermore, corynebacterium and Bacillus carnitus are present on the mucosa of FD patients but not in healthy persons, showing that the gut microbiota of FD patients differs from that of healthy people. Fermented milk containing Bifidobacteria alleviated postmeal discomfort and epigastric pain, implying a link between gut flora and FD.^[10]^

Current research suggests that gut microbiota is linked to FD, although whether the 2 are causally linked is uncertain. Two-sample Mendelian Randomization (TSMR)^[11,12]^ is a statistical method^[13]^ that studies the causality between exposure and outcome by using expose-associated genetic variation as a proxy for exposure and genetic variation randomly assigned at conception as an instrumental variable (IV) to estimate the causal effect of exposure on outcome. As a result, TSMR can reduce the impact of confounding circumstances.

In this study, we conducted TSMR research based on the summarized data of genome-wide association studies (GWAS) to investigate the potential causal relationship between gut microbiota and FD and to identify specific pathogenic flora, providing an innovative perspective for the study of FD: targeted regulation of specific bacterial groups to prevent and treat FD.

## 2. Methods

### 2.1. Study design

**Figure 1.**
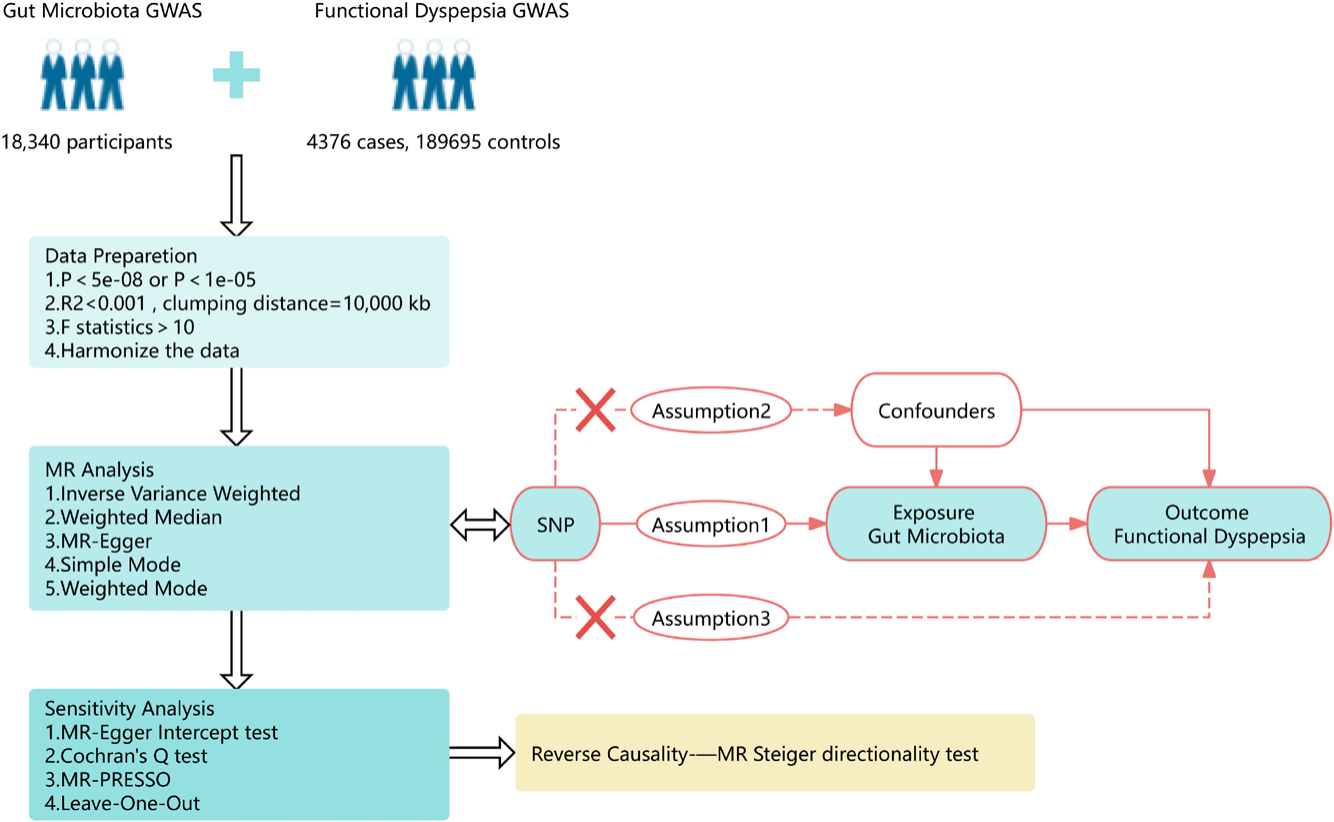
The study design of the associations of gut microbiota and functional dyspepsia. SNP = single nucleotide polymorphism.

The TSMR method was used to investigate the link between intestinal bacterial taxa and FD. Figure 1 depicts the overall design of this investigation. When performing TSMR analysis, 3 assumptions must be met in order to receive trustworthy results: (1) There is a strong correlation between genetic variation and exposure factors; (2) There is no correlation between genetic variation and confounding factors; (3) Genetic variation can only affect the outcome through exposure factors and not through other methods, implying that horizontal pleiotropy is not permitted. TSMR analysis can include genetic variations that satisfy these 3 hypotheses as IVs.^[11]^ Since the aggregate data utilized in this study are publicly available, participation in it does not require further ethical approval or consent.

### 2.2. Data sources

General statistical data on the gut microbiota^[14]^ from a large-scale GWAS MiBioGen alliance (http://MiBioGen.gcc.rug.nl). Of the 18,340 patients in the study, 24 were in the queue, and the majority (n = 13266) were of European descent. A total of 122,110 mutation sites were found. Genetic information on FD is derived from the IEU database (http://gwas.mrcieu.ac.uk/), which includes 189,695 controls and 4376 cases with European ancestry. The single nucleotide polymorphisms (SNPs) for this condition are 16380380.

### 2.3. Selection of instrumental variables

Following the exclusion of 15 gut microbiota without names, 196 bacterial types were included in the MR analysis. Following that, a series of quality control activities are performed to choose relevant IVs.

In this work, SNPs of significant significance to gut microbiota were preselected based on *P* *<* 5 × 10^‐8^, and it was discovered that only a small number of SNPs reached the threshold of genome-wide statistical significance. As a result, the threshold requirement^[15]^ was reduced, and SNPs with considerable relevance were chosen based on *P* *<* 1 × 10^‐5^.^[16,17]^ Set the linkage disequilibrium threshold at *R*^2^ < 0.001 and clumping distance = 10,000 kb to reduce the bias caused by residual genetic variation linkage imbalance. The *F* statistic, whose formula is $F=\frac{R^{2}}{1-R^{2}}\cdot \frac{N-k-1}{k}$, was used to assess the statistical strength of the correlation between SNPs and exposure. *N* is the sample size of the gut microbiota database, *K* is the number of SNPs, and *R*^2^ is the percentage of variation in gut microbiota explained by SNPs. We have $R^{2}=2\cdot \left(1-EAF\right)\cdot EAF\cdot \beta^{2}$ as the formula. Each SNP’s effect allele frequency is denoted by *EAF*, while the allele’s effect value is represented by *β*.^[11,17–20]^ *F* *<* 10 suggests a modest relationship between SNPs and exposure.^[21]^ To harmonize the effect sizes of exposure and outcome, the GWAS data of gut microbiota and SNPs with the same alleles in the GWAS results of FD were compiled.^[22]^

### 2.4. Statistical analysis

The causal connection between exposure (gut microbiota) and outcome (FD) was investigated using MR analysis. The primary analysis approach employed was inverse variance weighted (IVW) analysis, with auxiliary methods including weighted median, MR-Egger regression, simple mode, and weighted mode.^[23–25]^ The total estimate is obtained by combining the Wald estimates for each SNP using the IVW.^[26]^

### 2.5. Sensitivity analysis

Sensitivity analysis include horizontal pleiotropy, heterogeneity, and individual elimination. The MR-Egger regression test^[27]^ is used to assess horizontal pleiotropy. If the MR-Egger analysis shows a substantial intercept term, it suggests that the study has horizontal pleiotropy. SNP heterogeneity was determined using Cochran *Q* test.^[28]^ If the Cochran *Q* statistic test was statistically significant (*P* *<* .05), it demonstrated that there was significant heterogeneity in the analysis results. The MR pleiotropy residual sum and outlier test (MR-PRESSO)^[29]^ was used to identify outliers. If an outlier was found, it was eliminated and the remaining IVs were reanalyzed. To evaluate if a single SNP was responsible for any significant outcomes, leave-one analysis^[30]^ was employed. In FD, the association between human gut microbiota and the risk is represented by odds radio (OR) and its 95% confidence interval (CI). If *P* *<* .05, this suggests that there may be a causal association. All MR analysis is done in R (version 4.3.0) with the TSMR and MR-PRESSO packages.

## 3. Results

### 3.1. Instrumental variables selection

**Figure 2.**
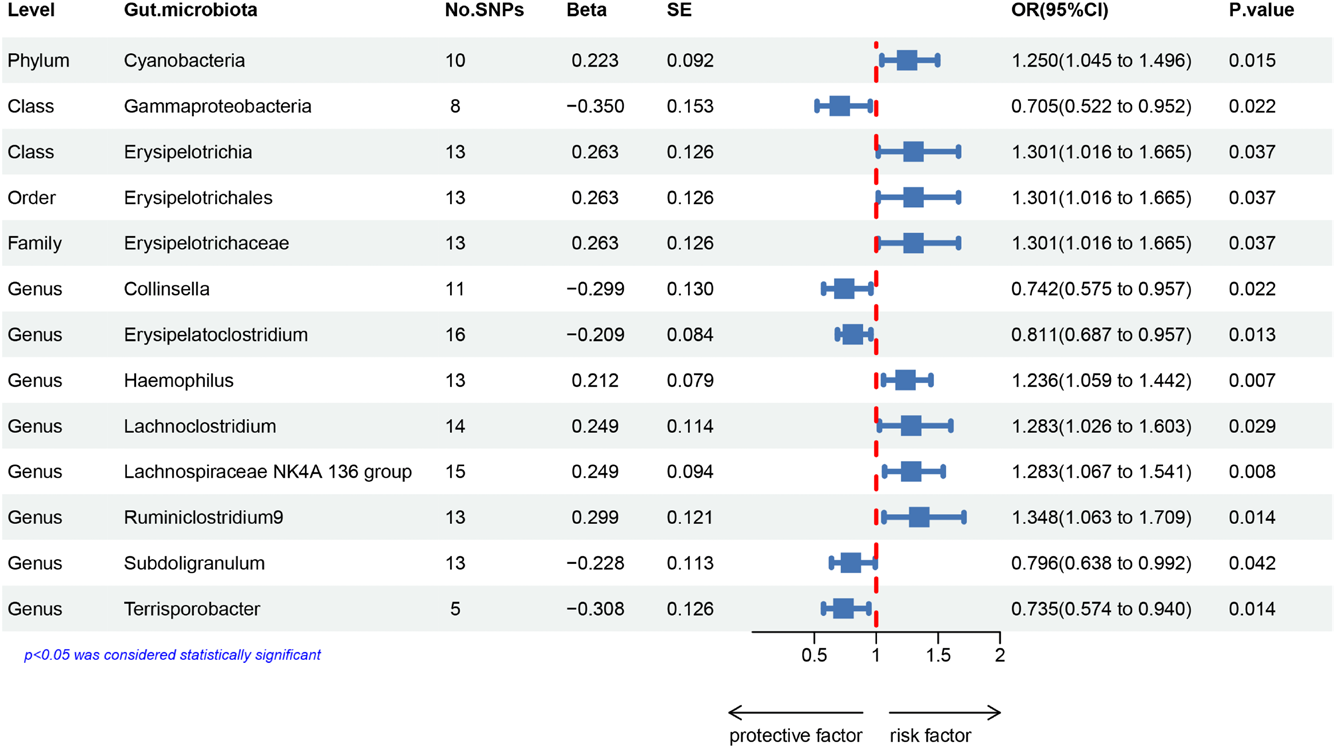
Forest plot depicting the associations between 13 genetically determined bacterial traits and the risk of functional dyspepsia. SNPs = number of single nucleotide polymorphisms, SE = standard error, CI = confidence interval, OR = odds ratio.

A total of 2817 independent SNPs (*P* *<* 1 × 10^‐5^) were related with 196 gut microbiota, including 5 taxonomic levels (phylum, class, order, family, and genus). Using the IVW approach in MR analysis, 11 bacterial traits linked to FD were isolated from the human gut microbiota (Fig. 2). By the end, 157 SNPs had been able to find (Supplementary Table S1, Supplemental Digital Content, http://links.lww.com/MD/N771). The F statistic is all more than 10, indicating that the bias of weak instrumental factors may be efficiently handled and avoided (Supplementary Table S1, Supplemental Digital Content, http://links.lww.com/MD/N771).

### 3.2. TSMR analysis

The IVW showed a positive correlation (OR: 1.250; 95% CI: 1.045, 1.496; *P* = .015) between the Phylum Cyanobacteria and the risk of FD. The Class Erysipelotrichia, Order Erysipelotrichales, and Family Erysipelotrichaceae were positively linked with the incidence of FD (OR: 1.301; 95% CI: 1.016, 1.665; *P* = .037). The Genus Haemophilus was found to be positively associated with the incidence of FD (OR: 1.236; 95% CI: 1.059, 1.442; *P* = .007). The Genus Lachnoclostridium was linked to an increased risk of FD (OR: 1.283; 95% CI: 1.026, 1.603; *P* = .029). The risk of FD was associated with the Genus Lachnospiraceae NK4A 136 group (OR: 1.283; 95% CI: 1.067, 1.541; *P* = .008). The Genus Ruminiclostridium 9 was linked to an increased risk of FD(OR: 1.348; 95% CI: 1.063, 1.709; *P* = .014).

On the other hand, FD risk was adversely correlated with Class Gammaproteobacteria (OR: 0.705; 95% CI: 0.522, 0.952; *P* = .022). The incidence of FD was negatively linked with the Genus Collinsella (OR: 0.742; 95% CI: 0.575, 0.957; *P* = .022). The Genus Erysipelatoclostridium was found to be adversely linked with the incidence of FD (OR: 0.811;95% CI:0.687, 0.957; *P* = .013). The Genus Subdoligranulum was related with a lower incidence of FD (OR: 0.796; 95% CI:0.638, 0.992; *P* = .042). The Genus Terrisporobacter was shown to be adversely associated with the incidence of FD (OR: 0.735; 95% CI: 0.574, 0.940; *P* = .014).

To further evaluate the potential of pleiotropic effects on causation, 31 SNPs were identified to be connected with confounding variables through the phenoscanner^[18]^ (http://www.phenoscanner.medschl.cam.ac.uk/) website query SNPs associated with this 13 bacteria, as indicated in Table 1. After being removed, these SNPs were recalculated using the MR Analysis technique. The Order Erysipelotrichales (OR: 1.301; 95% CI: 1.016, 1.665; *P* = .037), Family Erysipelotrichales (OR: 1.301; 95% CI: 1.016, 1.665; *P* = .037), Genus Haemophilus (OR: 1.236; 95% CI 1.059, 1.442; *P* = .007), Genus Ruminiclostridium 9 (OR: 1.422; 95% CI: 1.078, 1.877; *P* = .013), Genus Lachnospiraceae NK4A 136 group (OR: 1.297; 95% CI: 1.059, 1.589; *P* = .012), Class Gammaproteobacteria (OR: 0.705; 95% CI: 0.522, 0.952; *P* = .022), and Genus Erysipelatoclostridium (OR: 0.747; 95% CI: 0.628, 0.888; *P* = .001) and FD were all stable (Table 2). The results of the IVW analysis revealed that the Phylum Cyanobacteria, Class Erysipelotrichia, Genus Collinsella, Genus Lachnoclostridium, Genus Subdoligranulum, and Genus Terrisporobacter were unstable, as was FD.

**Table 1:** Details of the genetic variants with potential pleiotropy among instrumental variables used for gut microbiota.

Level	Gut microbiota	SNP	Trait	*P*-value
Phylum	Cyanobacteria	rs2585223	Height	3.28E‐09
		rs584122	MDC: %32+; mDC subset (CD32+)	1.51E‐06
		rs7148504	Coronary artery disease	2.79E‐10
Class	Erysipelotrichia	rs2300774	Mean corpuscular hemoglobin	3.72E‐83
		rs8003149	Impedance of arm left	2.15E‐11
Genus	Collinsella	rs11597285	Eosinophil percentage of white cells	4.57E‐11
		rs59414781	Diabetes diagnosed by doctor	5.33E‐06
	Erysipelatoclostridium	rs1434153	Home area population density: Scotland large urban area	3.40E‐06
		rs17804233	Plateletcrit	4.40E‐16
			Platelet count	3.68E‐11
			Platelet distribution width	3.84E‐09
		rs4697572	Impedance of leg left	2.30E‐06
	Lachnoclostridium	rs12566975	Hand grip strength left	5.49E‐07
		rs72829893	Trunk fat-free mass	2.83E‐07
		rs61915992	Comparative height size at age 10	7.34E‐08
		rs3821998	Eosinophil percentage of white cells	3.01E‐13
		rs6112314	Mean platelet volume	5.07E‐10
			Self-reported female infertility	9.11E-07
		rs4738679	Total cholesterol	3.35E‐12
	Lachnospiraceae NK4A136 group	rs7832116	Weight	1.51E‐07
		rs73044693	Cause of death: pneumonitis due to food and vomit	9.65E‐06
		rs7073658	Forced vital capacity	3.70E‐10
			Forced vital capacity, best measure	2.79E‐08
		rs4955932	Trans cis-18:2	5.55E‐06
	Ruminiclostridium 9	rs113048721	No results for rs113048721	0.00E+00
		rs12040548	Platelet count	5.05E‐22
			Mean platelet volume	1.52E‐19
		rs57665991	Forced vital capacity	9.09E‐16
		rs6082461	Self-reported gestational diabetes	6.95E‐06
	Subdoligranulum	rs10497836	Impedance of arm right	1.16E‐07
		rs4347804	Self-reported thalassemia	5.68E‐06
		rs2171249	Asthma 18 years old	8.45E‐06
		rs3761728	Weight	1.97E‐10
			Whole body fat-free mass	9.75E‐08
	Terrisporobacter	rs1883097	Heel bone mineral density	8.79E‐08
		rs7184125	Pulse rate	2.91E‐18
		rs2569953	Impedance of leg right	5.93E‐07

CD = cluster of differentiation, mDC = myeloid dendritic cell, MDC = myeloid dendritic cells.

**Table 2:** Summary of causality between gut microbiota and functional dyspepsia after correction.

Level	Gut microbiota	Nsnp	Methods	Beta	SE	OR (95%CI)	*P* value	Horizontal pleiotropy			Heterogeneity		Causal direction*	*F* statistic
								Egger intercept	SE	*P* value	Cochran *Q*	*P* value		
Class	Gammaproteobacteria	8	MR-Egger	‐0.597	0.497	0.550 (0.208–1.458)	.275	0.018	0.035	.620	4.573	0.600	TRUE	21.520
			Weighted median	‐0.395	0.201	0.674 (0.454–1.000)	.050							
			Inverse variance weighted	‐0.350	0.153	0.705 (0.522–0.952)	.022				4.846	0.679		
			Simple mode	‐0.493	0.340	0.611 (0.314–1.188)	.190							
			Weighted mode	‐0.537	0.317	0.585 (0.314–1.087)	.134							
Family	Erysipelotrichaceae	13	MR-Egger	0.096	0.567	1.100 (0.362–3.344)	.869	0.010	0.034	.767	11.840	0.376	TRUE	21.711
			Weighted median	0.141	0.175	1.151 (0.817–1.622)	.421							
			Inverse variance weighted	0.263	0.126	1.301 (1.016–1.665)	.037				11.939	0.451		
			Simple mode	0.072	0.263	1.075 (0.642–1.801)	.788							
			Weighted mode	0.072	0.271	1.075 (0.632–1.828)	.794							
Genus	Erysipelatoclostridium	13	MR-Egger	‐0.470	0.334	0.625 (0.325–1.203)	.187	0.015	0.028	.592	10.750	0.464	TRUE	22.008
			Weighted median	‐0.329	0.123	0.720 (0.565–0.917)	.008							
			Inverse variance weighted	‐0.292	0.088	0.747 (0.628–0.888)	.001				11.054	0.524		
			Simple mode	‐0.384	0.213	0.681 (0.449–1.033)	.096							
			Weighted mode	‐0.392	0.231	0.676 (0.430–1.064)	.116							
Genus	Haemophilus	13	MR-Egger	0.066	0.195	1.068 (0.728–1.566)	.744	0.017	0.021	.429	11.275	0.421	TRUE	22.919
			Weighted median	0.229	0.113	1.258 (1.008–1.570)	.043							
			Inverse variance weighted	0.212	0.079	1.236 (1.059–1.442)	.007				11.964	0.449		
			Simple mode	0.366	0.190	1.442 (0.994–2.094)	.078							
			Weighted mode	0.259	0.176	1.296 (0.918–1.827)	.166							
Genus	Lachnospiraceae NK4A 136 group	11	MR-Egger	0.271	0.194	1.311 (0.897–1.917)	.196	‐0.001	0.015	.950	6.611	0.678	TRUE	21.509
			Weighted median	0.251	0.148	1.286 (0.961–1.719)	.090							
			Inverse variance weighted	0.260	0.104	1.297 (1.059–1.589)	.012				6.616	0.761		
			Simple mode	0.171	0.225	1.187 (0.764–1.843)	.464							
			Weighted mode	0.299	0.170	1.349 (0.968–1.881)	.108							
Genus	Ruminiclostridium 9	9	MR-Egger	1.009	0.665	2.743 (0.745–10.102)	.173	‐0.047	0.047	.346	6.423	0.491	TRUE	21.322
			Weighted median	0.319	0.191	1.376 (0.947–2.000)	.094							
			Inverse variance weighted	0.352	0.141	1.422 (1.078–1.877)	.013				7.444	0.490		
			Simple mode	0.505	0.299	1.658 (0.922–2.979)	.130							
			Weighted mode	0.342	0.276	1.408 (0.819–2.419)	.250							
Order	Erysipelotrichales	13	MR-Egger	0.096	0.567	1.100 (0.362–3.344)	.869	0.010	0.034	.767	11.840	0.376	TRUE	21.711
			Weighted median	0.141	0.173	1.151 (0.820–1.615)	.415							
			Inverse variance weighted	0.263	0.126	1.301 (1.016–1.665)	.037				11.939	0.451		
			Simple mode	0.072	0.268	1.075 (0.636–1.817)	.792							
			Weighted mode	0.072	0.277	1.075 (0.625–1.850)	.799							

CI = confidence interval, MR = Mendelian randomization, OR = odds ratio, SE = standard error, SNP = single nucleotide polymorphism.

* The “TRUE” results concerning the causal direction mean there is no reverse causation.

### 3.3. Sensitivity analysis and reverse causality

Using MR-Egger regression, we evaluated the horizontal pleiotropy between SNPs and FD. The analysis showed no evidence of horizontal pleiotropy, as indicated by the intercept *P*-values for various taxa: the Class Gammaproteobacteria (*P* = .620), the Order Erysipelotrichales (*P* = .767), the Family Erysipelotrichaceae (*P* = .767), the Genus Erysipelatoclostridium (*P* = .592), the Genus Ruminiclostridium 9 (*P* = .346), and the Genus Lachnospiraceae NK4A 136 group (*P* = .950). Additionally, MR-Egger regression intercepts for the Genus Haemophilus (*P* = .429) were >0.05. These results are detailed in Table 2.

**Figure 3.**
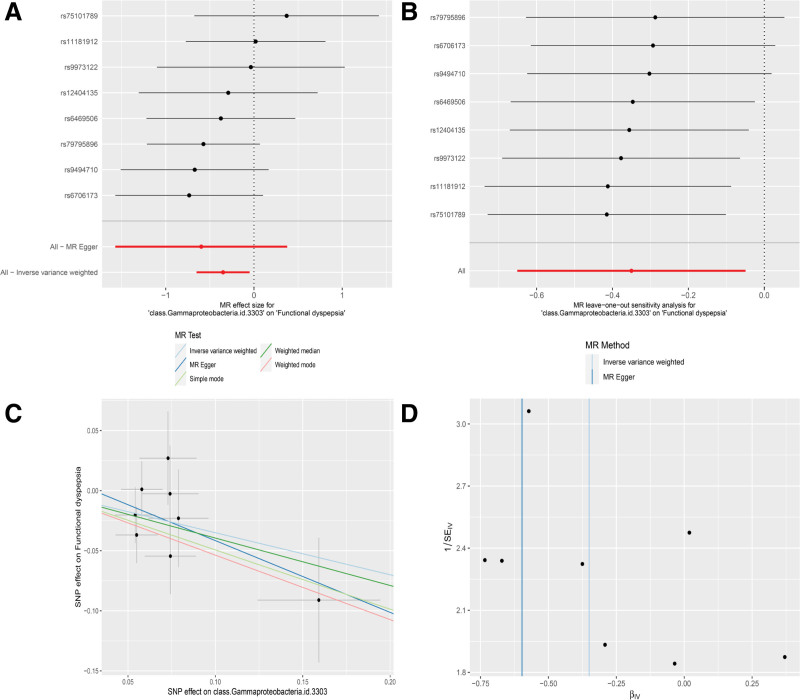
Forest plot (A), sensitivity analysis (B), scatter plot (C), and funnel plot (D) of the causal effect of Class Gammaproteobacteria on functional dyspepsia.

The Cochran test was employed to evaluate the heterogeneity of SNPs (Table 2). The results indicated no significant heterogeneity across the studied taxa—Class Gammaproteobacteria, Order Erysipelotrichales, Family Erysipelotrichaceae, and the Genus Erysipelatoclostridium, Ruminiclostridium 9, Lachnospiraceae NK4A 136 group, and Haemophilus—with all *Q* values from IVW and MR-Egger analyses exceeding 0.05. Furthermore, the MR-PRESSO method detected no outliers, as all *P*-values were above .05 (Table 2). These findings corroborate the causal association between FD and the mentioned organisms: Class Gammaproteobacteria (Fig. 3), Order Erysipelotrichales (Supplementary Figure S1, Supplemental Digital Content, http://links.lww.com/MD/N771), Family Erysipelotrichaceae (Supplementary Figure S2, Supplemental Digital Content, http://links.lww.com/MD/N771), and the Genus Erysipelatoclostridium (Supplementary Figure S3, Supplemental Digital Content, http://links.lww.com/MD/N771), Ruminiclostridium 9 (Supplementary Figure S4, Supplemental Digital Content, http://links.lww.com/MD/N771), Lachnospiraceae NK4A 136 group (Supplementary Figure S5, Supplemental Digital Content, http://links.lww.com/MD/N771), and Haemophilus (Supplementary Figure S6, Supplemental Digital Content, http://links.lww.com/MD/N771).

To further explore whether there is a reverse causal relationship between gut microbiota and FD, we employed the MR Steiger directionality test.^[31]^ The test results showed no reverse causal effect between gut microbiota and FD, further strengthening the hypothesis of a direct relationship between the exposure factors and outcome variables (Table 2).

## 4. Discussion

In our TSMR study, thirteen gut microbiota were found to be associated with FD. These taxa include Phylum Cyanobacteria, Class Erysipelotrichia, Order Erysipelotrichales, Family Erysipelotrichaceae, and the Genus Haemophilus, Lachnoclostridium, Lachnospiraceae NK4A 136 group, Ruminiclostridium 9, Gammaproteobacteria, Collinsella, Erysipelatoclostridium, Subdoligranulum, and Terrisporobacter. To mitigate the effects of confounding variables, SNP quality was rigorously monitored. Seven of these taxa—Order Erysipelotrichales, Family Erysipelotrichaceae, and the Genus Haemophilus, Ruminiclostridium 9, Lachnospiraceae NK4A 136 group, Gammaproteobacteria, and Erysipelatoclostridium—have been identified to causally influence the risk of FD.

The human gastrointestinal tract comprises a complex ecosystem of symbiotic microbial communities. These gut microbiota play a crucial role in maintaining human health by co-metabolizing substances with the host and regulating the dynamic balance of intestinal microecology. Structural changes in the gut microbiota can influence the host’s physiological and pathological states.^[32]^ Numerous studies^[33,34]^ have demonstrated that dysbiosis of the gut microbiota may induce low-grade mucosal inflammation (marked by slight eosinophil and mast cell degranulation), activate the systemic immune system, and compromise the integrity and permeability of the intestinal barrier. Such disruptions may also affect the microbial–gut–brain axis, potentially initiating and exacerbating FD. Once this equilibrium is disturbed, the ecological imbalance of the gut microbiota can lead to various pathogenic changes.

Risk factors for FD include various microbial taxa such as Ruminiclostridium 9, Haemophilus, the Lachnospiraceae NK4A 136 group, the Order Erysipelotrichales, and the Family Erysipelotrichaceae. Among these, 4 belong to the phylum Firmicutes, while Haemophilus is a member of Proteobacteria. High-throughput 16S rDNA sequencing in a study^[35]^ of FD model rats with Liver Depression-Spleen Deficiency Syndrome showed a significant decrease in the relative abundance of Bacteroides and an increase in Proteobacteria and Firmicutes in the fecal samples of FD rats compared to healthy controls. Moreover, a high-protein, high-calorie, and high-fat diet used to induce dyspepsia in mice significantly disturbed the gut microbiota and elevated the abundance of the Lachnospiraceae NK4A 136 group.^[36]^ Additionally, the Jiao Sanxian decoction appeared to mitigate the enrichment of the Lachnospiraceae NK4A 136 group in the model rats compared to the control group.^[37]^

The Genus Erysipelotrichales, initially identified by Verbarg S^[38]^ in the digestive tracts of animals^[39]^ and insects,^[40]^ has been associated with gastrointestinal disorders^[41]^ and host lipid metabolism. Subsequently, an increase in the relative abundance of Erysipelotrichaceae was observed in rat models afflicted with gastrointestinal disorders.^[42]^ Previous research^[43]^ found that patients with dyspepsia exhibited higher concentrations of Haemophilus and Rothia in their duodenums. These studies suggest that elevated levels of the Lachnospiraceae NK4A 136 group, Ruminiclostridium 9, Haemophilus, the Order Erysipelotrichales, and the Family Erysipelotrichaceae may correlate positively with the risk of FD, corroborating our findings.

TSMR analysis suggests that the Genus Erysipelatoclostridium and the Class Gammaproteobacteria may act as protective agents against FD. Erysipelatoclostridium, a Gram-positive anaerobic bacterium formerly known as *Clostridium ramosum*, was renamed in 2013.^[44]^ Gammaproteobacteria, part of the phylum Proteobacteria,^[45]^ is categorized into 5 subgroups: alpha, beta, gamma, delta, and epsilon.^[46]^ However, no studies have yet been published on changes in Gammaproteobacteria and Erysipelatoclostridium levels in FD patients. The abundance of Erysipelatoclostridium in the small intestine correlates positively with the concentration of glucose and fat transporters.^[47]^ Additionally, research indicates a significant increase in short-chain fatty acids in fecal samples when the level of Gammaproteobacteria in fetuses from obese mothers was reduced.^[48]^ Given that our study is the first to characterize the unique gut microbiota associated with FD, it may provide new insights into the mechanisms underlying this condition. The relationship between these bacterial species and FD remains poorly understood.

Previous research has consistently shown that disruptions in the gut microbiota, characterized by changes in the number, relative abundance, and composition of species, are common among individuals with FD. Additionally, substantial evidence supports the safe and effective modulation of gut microbiota by probiotics in treating FD.^[49–51]^ This study is the first to demonstrate a genetically-based causal relationship between gut microbiota and FD using TSMR experiments. By applying stringent criteria for IVs and eliminating confounding factors, 7 bacterial taxa associated with FD were identified. Our MR analysis sheds light on the role of gut microbiota in the pathophysiology of FD and provides a new perspective for FD research, emphasizing the management of specific bacterial groups to enhance beneficial bacteria and inhibit harmful ones.

Our study does have several limitations. First, the generalizability of our findings to other racial or ethnic groups may be limited because the patient and control groups were predominantly European. Second, our analysis of the gut microbiota was confined to the genus level; we did not assess species or strain-specific variations. Third, further research is required to identify the specific subtypes of gut microbiota associated with FD, which were not examined in this study.

## 5. Conclusions

This study comprehensively investigated the potential causative role of gut microbiota in FD. The research suggests that FD may be causally associated with several taxa, including the Class Gammaproteobacteria, the Order Erysipelotrichales, the Family Erysipelotrichaceae, and the Genus Haemophilus, Ruminiclostridium 9, Lachnospiraceae NK4A 136 group, and Erysipelatoclostridium. These findings provide new insights into potential therapeutic targets for FD and illuminate aspects of the disease’s etiology.

## Acknowledgments

We acknowledge the participants and investigators of the MiBioGen and IEU studies.

## Author contributions

**Conceptualization:** Xiaojing Jin, Keli Xu.

**Data curation:** Xiaojing Jin, Keli Xu, Jingyi Wu, Chenxi Yang.

**Formal analysis:** Xiaojing Jin, Keli Xu, Jingyi Wu, Chenxi Yang, Chuanlong Zhou.

**Methodology:** Xiaojing Jin, Keli Xu, Chuanlong Zhou.

**Writing – original draft:** Xiaojing Jin, Keli Xu, Jingyi Wu, Chenxi Yang.

**Writing – review & editing:** Xiaojing Jin, Keli Xu, Jie Bao, Lijun Du, Binrui Chen, Xiaomei Shao, Chuanlong Zhou.

## Supplementary Material


