# Supplementary material for: The causality between gut microbiota and functional dyspepsia: A two-sample Mendelian randomization analysis
Source: Medicine (Baltimore). 2024 Oct 25;103(43):e40180. doi: 10.1097/MD.0000000000040180 (PMC11521013; doi:10.1097/MD.0000000000040180)
Supplement: Supplementary file 1 [file medi-103-e40180-s001.docx]

Supplement figure and Table


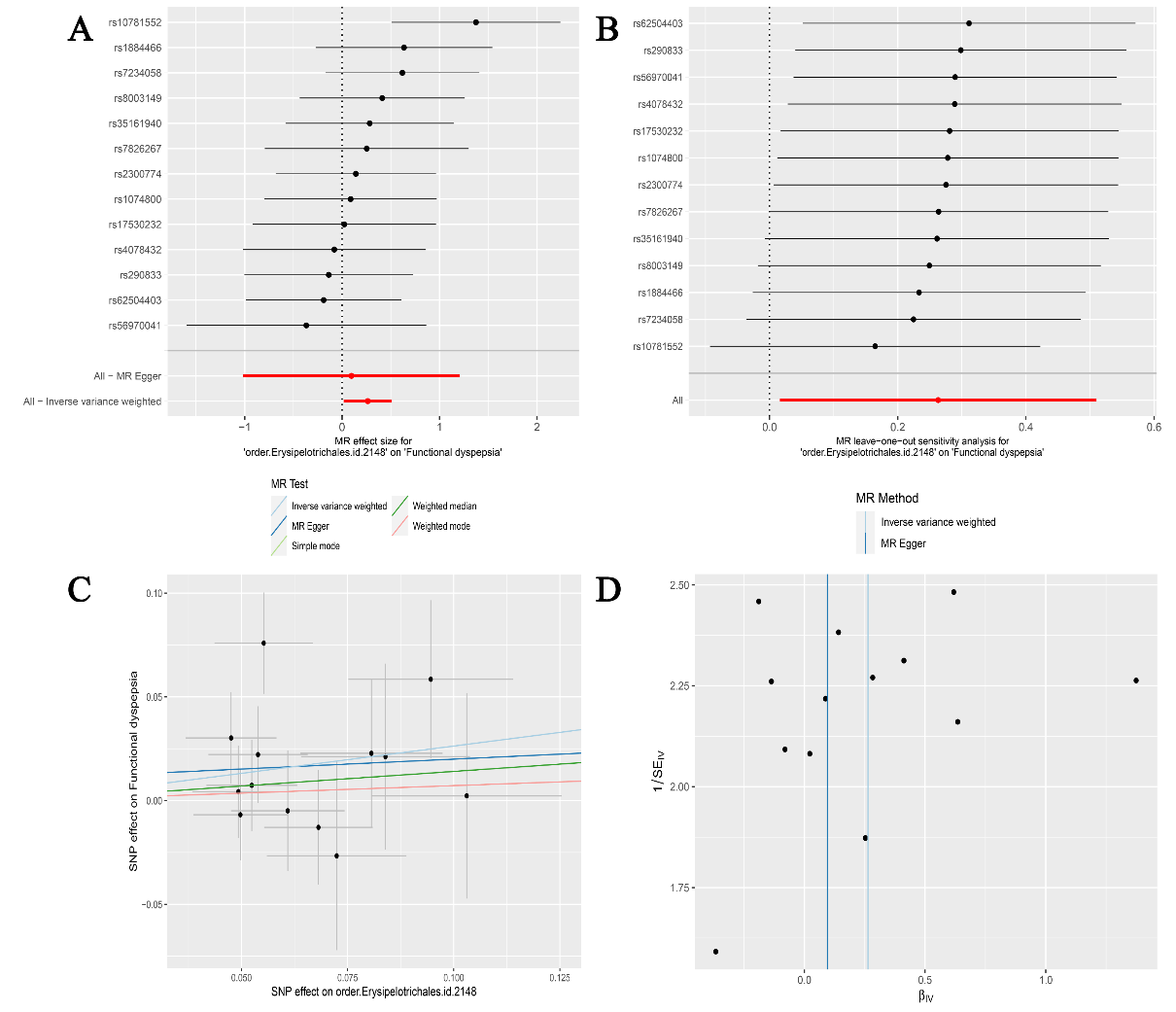


Figure S1


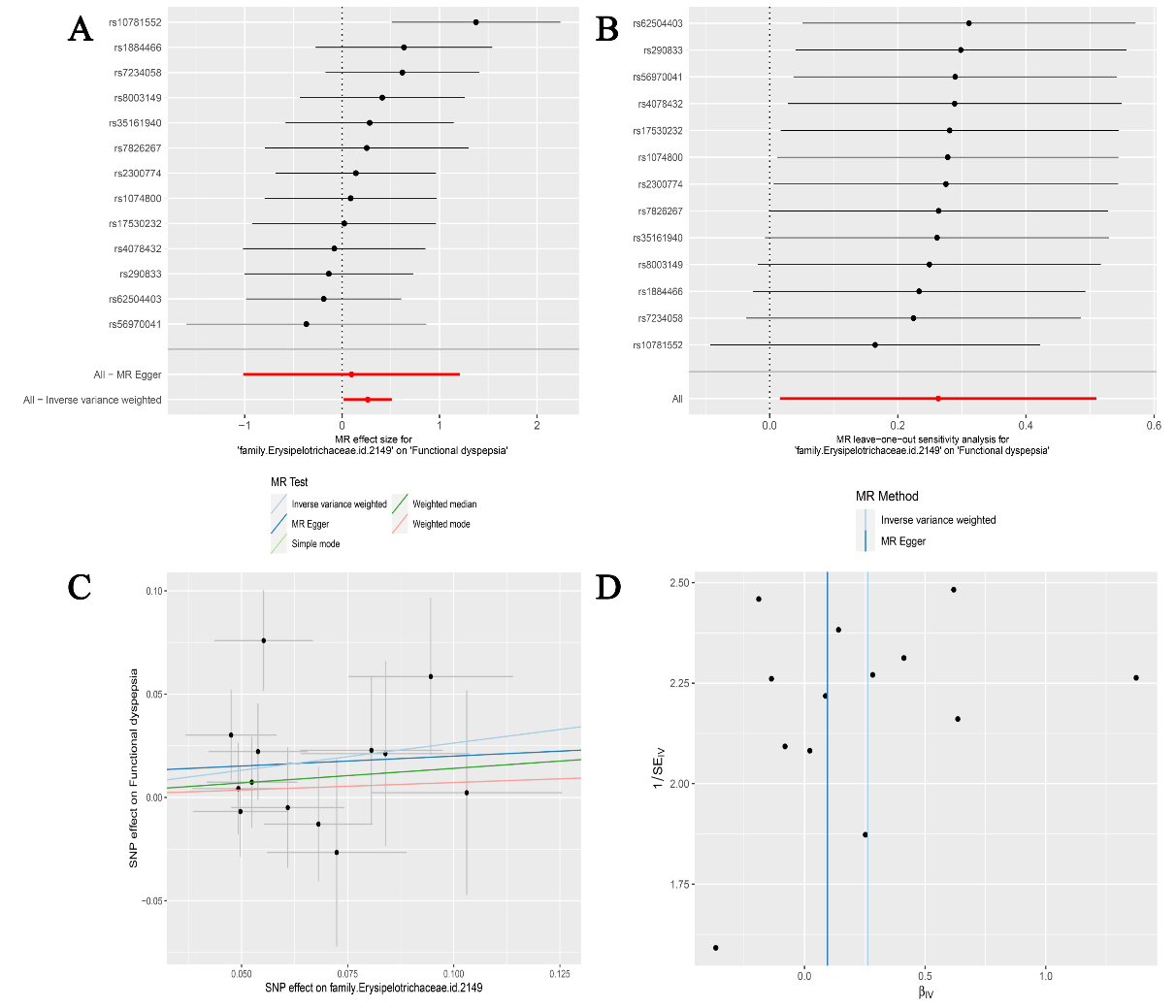


Figure S2


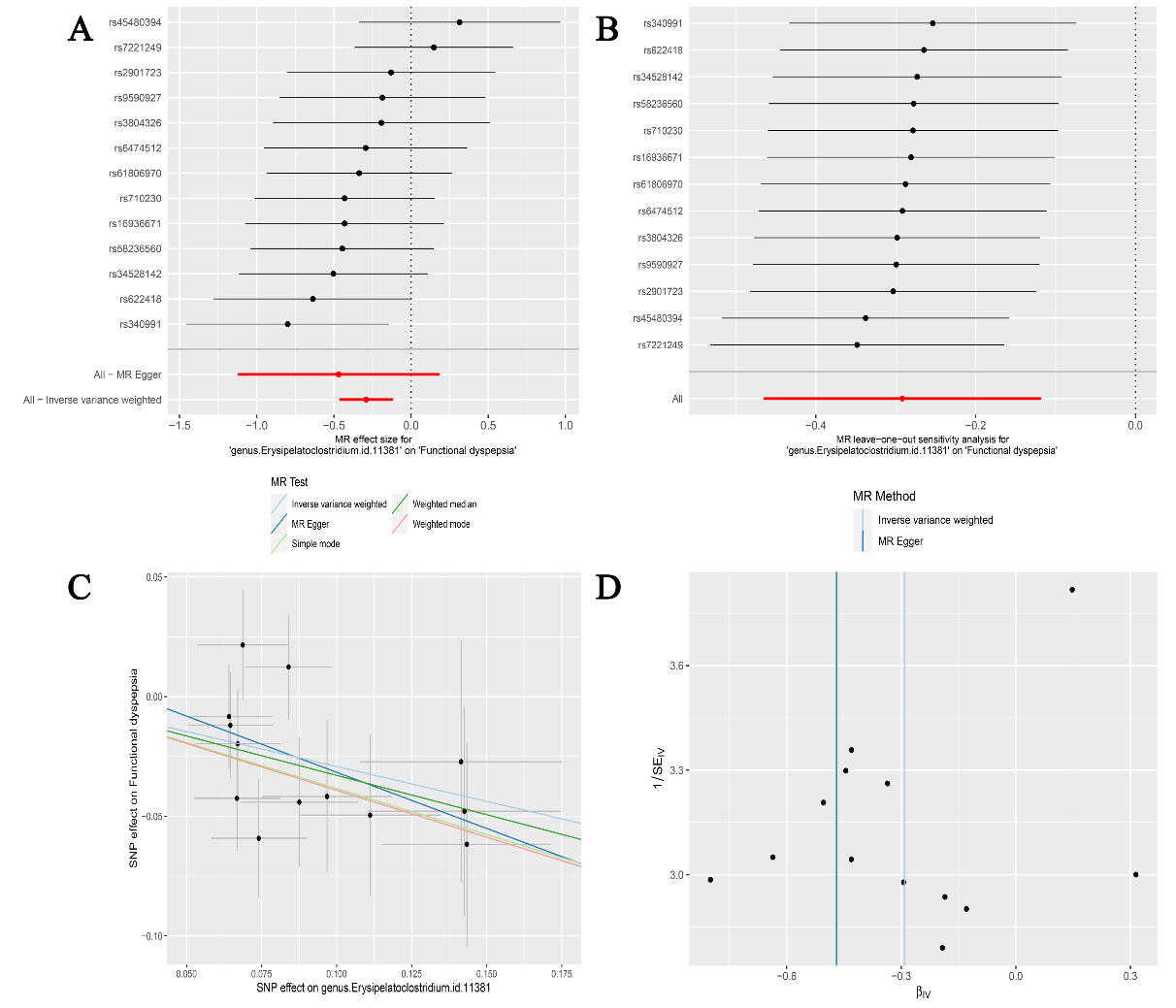


Figure S3


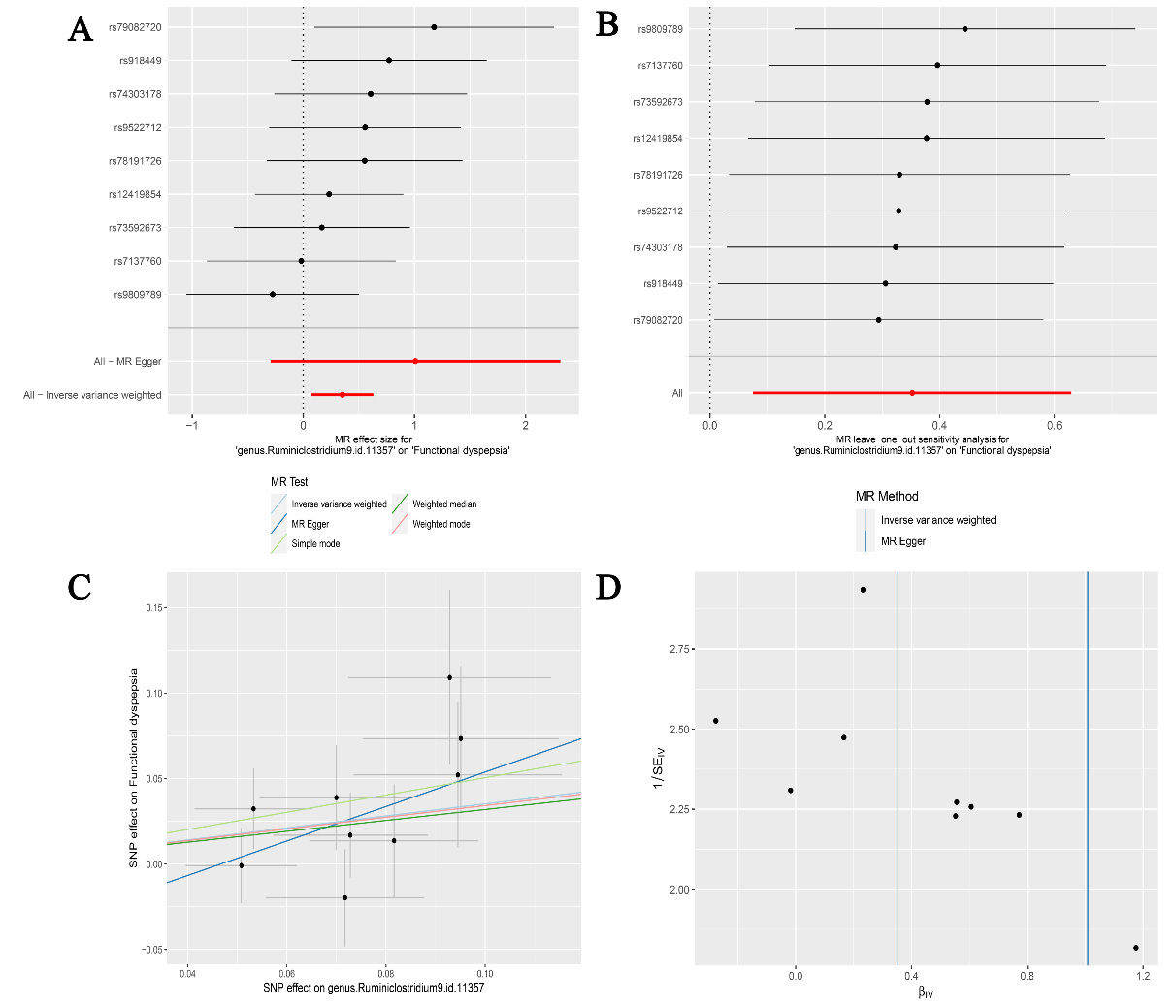


Figure S4


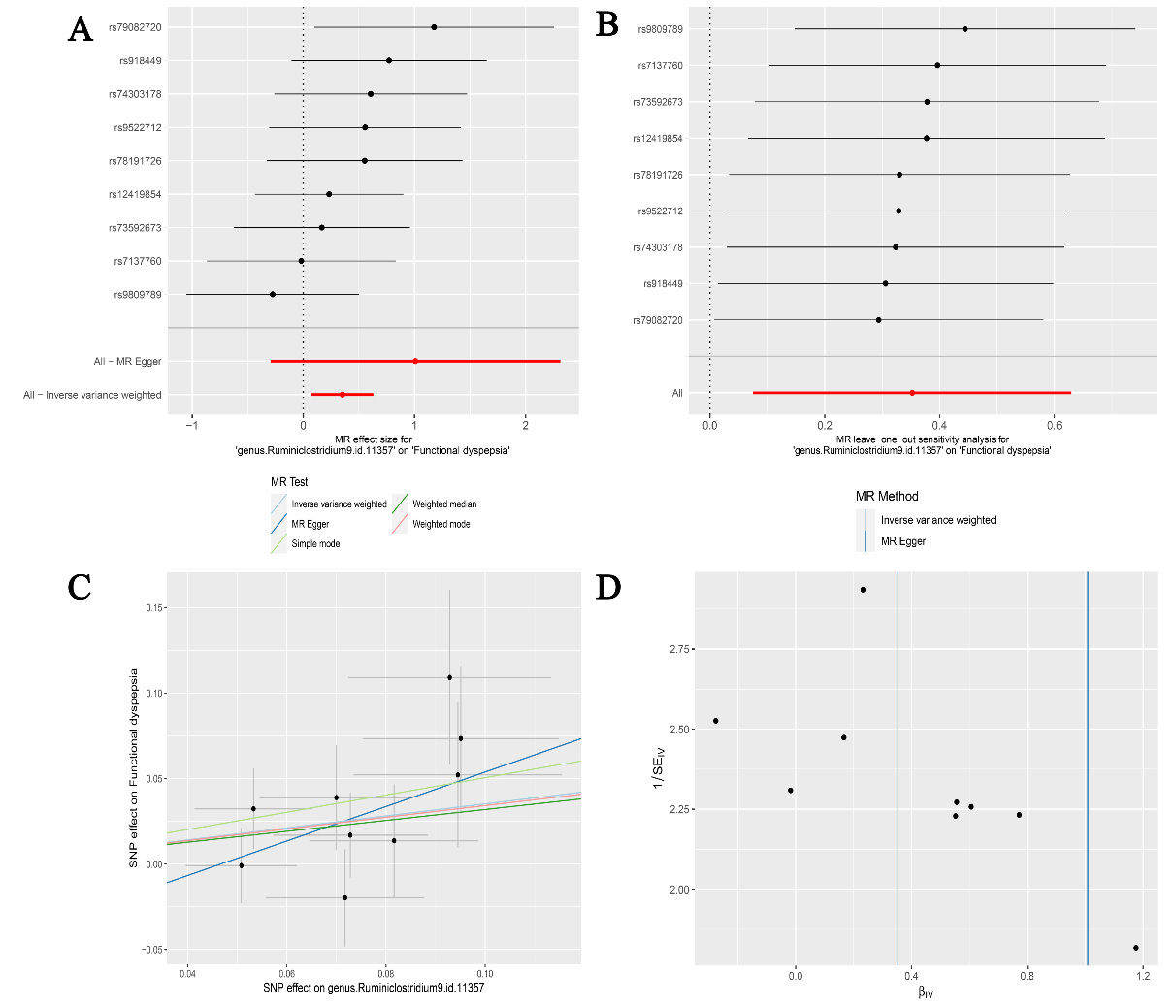


Figure S5


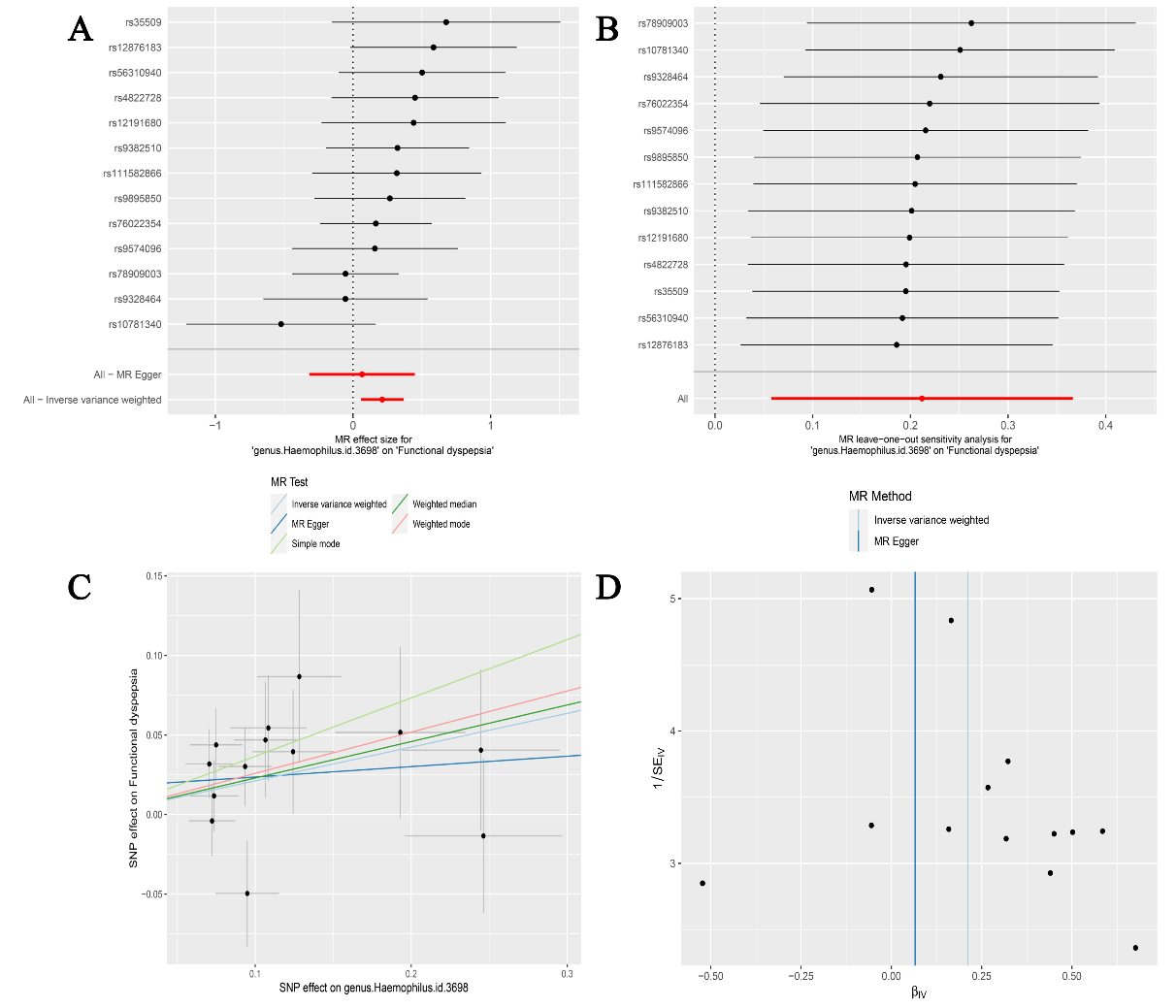


Figure S6

Table S1

| **Supplementary Table 1: Detailed information on instrumental variables used in the MR analyses** | | | | | | | | | | |
| --- | --- | --- | --- | --- | --- | --- | --- | --- | --- | --- |
| **Bacterial traits** | **SNP** | **Effect allele** | **Other allele** | **Gut microbiota** | | | **FD** | | | **F** |
|  |  |  |  | **Beta** | **Se** | **P value** | **Beta** | **Se** | **P value** |  |
| phylum.Cyanobacteria | rs9864379 | T | C | -0.139 | 0.027 | 2.03E-07 | 0.002 | 0.031 | 0.953 | 21.040 |
| phylum.Cyanobacteria | rs789068 | G | A | -0.111 | 0.021 | 1.57E-07 | -0.076 | 0.031 | 0.015 | 20.660 |
| phylum.Cyanobacteria | rs76531781 | T | C | -0.232 | 0.049 | 2.87E-06 | -0.091 | 0.056 | 0.101 | 20.721 |
| phylum.Cyanobacteria | rs7148504 | G | T | 0.08 | 0.018 | 6.62E-06 | 0.024 | 0.022 | 0.276 | 25.935 |
| phylum.Cyanobacteria | rs61972390 | T | C | 0.107 | 0.024 | 9.11E-06 | -0.026 | 0.032 | 0.427 | 28.282 |
| phylum.Cyanobacteria | rs584122 | C | T | -0.152 | 0.033 | 4.23E-06 | -0.012 | 0.047 | 0.796 | 23.610 |
| phylum.Cyanobacteria | rs2585223 | T | C | 0.111 | 0.025 | 8.86E-06 | 0.023 | 0.034 | 0.484 | 27.049 |
| phylum.Cyanobacteria | rs2553290 | A | T | 0.096 | 0.02 | 3.61E-06 | 0.01 | 0.031 | 0.749 | 21.887 |
| phylum.Cyanobacteria | rs2314810 | C | G | -0.218 | 0.046 | 4.25E-06 | -0.018 | 0.052 | 0.737 | 20.480 |
| phylum.Cyanobacteria | rs12555298 | G | A | 0.097 | 0.022 | 8.09E-06 | 0.069 | 0.029 | 0.017 | 23.741 |
| order.Erysipelotrichales | rs8003149 | C | T | 0.054 | 0.012 | 4.08E-06 | 0.022 | 0.023 | 0.34 | 23.764 |
| order.Erysipelotrichales | rs7826267 | T | G | -0.084 | 0.02 | 9.28E-06 | -0.021 | 0.045 | 0.635 | 21.040 |
| order.Erysipelotrichales | rs7234058 | T | C | -0.095 | 0.019 | 9.12E-07 | -0.059 | 0.038 | 0.125 | 19.975 |
| order.Erysipelotrichales | rs62504403 | C | T | 0.068 | 0.013 | 1.12E-07 | -0.013 | 0.028 | 0.64 | 20.476 |
| order.Erysipelotrichales | rs56970041 | T | G | 0.072 | 0.016 | 5.40E-06 | -0.027 | 0.046 | 0.559 | 26.961 |
| order.Erysipelotrichales | rs4078432 | C | T | -0.061 | 0.013 | 4.23E-06 | 0.005 | 0.029 | 0.866 | 20.142 |
| order.Erysipelotrichales | rs35161940 | T | C | -0.081 | 0.017 | 1.85E-06 | -0.023 | 0.036 | 0.521 | 20.186 |
| order.Erysipelotrichales | rs290833 | T | G | -0.05 | 0.011 | 8.03E-06 | 0.007 | 0.022 | 0.757 | 20.433 |
| order.Erysipelotrichales | rs2300774 | G | A | 0.052 | 0.011 | 8.95E-07 | 0.007 | 0.022 | 0.736 | 23.186 |
| order.Erysipelotrichales | rs1884466 | C | T | -0.048 | 0.011 | 9.53E-06 | -0.03 | 0.022 | 0.171 | 22.501 |
| order.Erysipelotrichales | rs17530232 | A | G | 0.103 | 0.022 | 2.79E-06 | 0.002 | 0.05 | 0.963 | 20.613 |
| order.Erysipelotrichales | rs10781552 | C | T | -0.055 | 0.012 | 2.33E-06 | -0.076 | 0.024 | 0.002 | 26.858 |
| order.Erysipelotrichales | rs1074800 | A | G | 0.049 | 0.011 | 6.15E-06 | 0.004 | 0.022 | 0.846 | 22.060 |
| genus.Terrisporobacter | rs7184125 | T | C | 0.091 | 0.021 | 8.48E-06 | -0.027 | 0.025 | 0.269 | 34.612 |
| genus.Terrisporobacter | rs58405430 | G | T | 0.135 | 0.03 | 7.94E-06 | -0.05 | 0.046 | 0.285 | 21.735 |
| genus.Terrisporobacter | rs2872237 | C | A | -0.081 | 0.018 | 3.97E-06 | 0.034 | 0.022 | 0.133 | 19.794 |
| genus.Terrisporobacter | rs2569953 | A | C | -0.078 | 0.017 | 8.95E-06 | 0.01 | 0.022 | 0.657 | 21.730 |
| genus.Terrisporobacter | rs1883097 | C | T | 0.226 | 0.045 | 4.16E-07 | -0.077 | 0.058 | 0.183 | 22.702 |
| genus.Subdoligranulum | rs76664262 | T | A | 0.083 | 0.019 | 4.87E-06 | -0.03 | 0.035 | 0.389 | 21.245 |
| genus.Subdoligranulum | rs76528319 | G | T | -0.143 | 0.031 | 7.41E-06 | 0.046 | 0.04 | 0.246 | 20.457 |
| genus.Subdoligranulum | rs75158211 | T | C | -0.072 | 0.016 | 7.52E-06 | -0.001 | 0.031 | 0.984 | 23.654 |
| genus.Subdoligranulum | rs6555306 | T | C | -0.074 | 0.016 | 2.81E-06 | -0.015 | 0.031 | 0.628 | 21.714 |
| genus.Subdoligranulum | rs4347804 | A | G | 0.166 | 0.036 | 2.18E-06 | -0.015 | 0.062 | 0.811 | 20.037 |
| genus.Subdoligranulum | rs3761728 | T | G | -0.054 | 0.012 | 3.87E-06 | 0.006 | 0.025 | 0.829 | 22.779 |
| genus.Subdoligranulum | rs35940633 | G | A | -0.051 | 0.011 | 4.22E-06 | 0.001 | 0.023 | 0.959 | 22.196 |
| genus.Subdoligranulum | rs2171249 | C | T | 0.107 | 0.023 | 4.51E-06 | -0.071 | 0.043 | 0.097 | 19.948 |
| genus.Subdoligranulum | rs2114677 | C | T | -0.104 | 0.023 | 2.72E-06 | -0.002 | 0.034 | 0.961 | 20.187 |
| genus.Subdoligranulum | rs16962433 | A | T | 0.086 | 0.019 | 7.65E-06 | -0.074 | 0.045 | 0.096 | 21.574 |
| genus.Subdoligranulum | rs1667315 | G | A | 0.049 | 0.011 | 6.72E-06 | -0.023 | 0.022 | 0.307 | 20.372 |
| genus.Subdoligranulum | rs10497836 | C | T | -0.052 | 0.012 | 8.38E-06 | 0.061 | 0.027 | 0.022 | 19.725 |
| genus.Subdoligranulum | rs10065321 | T | C | -0.051 | 0.011 | 2.10E-06 | -0.02 | 0.022 | 0.377 | 19.594 |
| genus.Ruminiclostridium9 | rs9809789 | C | T | -0.072 | 0.016 | 8.72E-06 | 0.02 | 0.028 | 0.485 | 24.091 |
| genus.Ruminiclostridium9 | rs9522712 | T | C | 0.07 | 0.015 | 4.66E-06 | 0.039 | 0.031 | 0.207 | 20.457 |
| genus.Ruminiclostridium9 | rs918449 | A | G | -0.095 | 0.02 | 2.56E-06 | -0.073 | 0.043 | 0.085 | 19.940 |
| genus.Ruminiclostridium9 | rs79082720 | C | G | 0.093 | 0.02 | 6.47E-06 | 0.109 | 0.051 | 0.033 | 20.930 |
| genus.Ruminiclostridium9 | rs78191726 | T | C | 0.094 | 0.021 | 7.58E-06 | 0.052 | 0.042 | 0.219 | 23.116 |
| genus.Ruminiclostridium9 | rs74303178 | T | C | 0.053 | 0.012 | 7.92E-06 | 0.032 | 0.024 | 0.17 | 21.860 |
| genus.Ruminiclostridium9 | rs73592673 | A | T | -0.082 | 0.017 | 2.14E-06 | -0.014 | 0.033 | 0.681 | 20.499 |
| genus.Ruminiclostridium9 | rs7137760 | C | T | 0.051 | 0.011 | 7.07E-06 | -0.001 | 0.022 | 0.966 | 24.635 |
| genus.Ruminiclostridium9 | rs6082461 | A | C | 0.059 | 0.013 | 4.87E-06 | -0.029 | 0.027 | 0.29 | 21.427 |
| genus.Ruminiclostridium9 | rs57665991 | C | G | -0.064 | 0.012 | 2.07E-07 | -0.042 | 0.026 | 0.101 | 21.798 |
| genus.Ruminiclostridium9 | rs12419854 | T | A | -0.073 | 0.016 | 3.18E-06 | -0.017 | 0.025 | 0.495 | 21.040 |
| genus.Ruminiclostridium9 | rs12040548 | G | T | 0.057 | 0.012 | 3.15E-06 | 0.024 | 0.025 | 0.321 | 22.630 |
| genus.Ruminiclostridium9 | rs113048721 | C | G | 0.06 | 0.013 | 4.10E-06 | -0.012 | 0.031 | 0.694 | 20.721 |
| genus.LachnospiraceaeNK4A136group | rs954878 | A | G | -0.052 | 0.011 | 1.78E-06 | -0.042 | 0.023 | 0.067 | 21.245 |
| genus.LachnospiraceaeNK4A136group | rs7832116 | A | G | -0.071 | 0.015 | 3.57E-06 | -0.035 | 0.033 | 0.284 | 28.367 |
| genus.LachnospiraceaeNK4A136group | rs76193507 | A | G | -0.23 | 0.05 | 2.93E-06 | -0.084 | 0.039 | 0.033 | 19.383 |
| genus.LachnospiraceaeNK4A136group | rs7616165 | G | T | -0.231 | 0.048 | 2.77E-06 | -0.008 | 0.07 | 0.911 | 17.753 |
| genus.LachnospiraceaeNK4A136group | rs73044693 | A | G | -0.108 | 0.023 | 3.57E-06 | -0.017 | 0.044 | 0.696 | 19.696 |
| genus.LachnospiraceaeNK4A136group | rs7073658 | T | G | -0.05 | 0.011 | 5.27E-06 | 0.013 | 0.022 | 0.563 | 21.752 |
| genus.LachnospiraceaeNK4A136group | rs68104925 | T | C | -0.055 | 0.012 | 2.37E-06 | -0.038 | 0.024 | 0.11 | 21.129 |
| genus.LachnospiraceaeNK4A136group | rs59805249 | T | C | 0.094 | 0.021 | 9.45E-06 | 0.014 | 0.038 | 0.716 | 22.762 |
| genus.LachnospiraceaeNK4A136group | rs4955932 | T | C | -0.049 | 0.011 | 7.05E-06 | -0.022 | 0.023 | 0.336 | 23.091 |
| genus.LachnospiraceaeNK4A136group | rs2880566 | T | C | 0.06 | 0.013 | 5.61E-06 | -0.016 | 0.031 | 0.598 | 20.258 |
| genus.LachnospiraceaeNK4A136group | rs28540839 | A | C | 0.051 | 0.011 | 9.34E-06 | 0.012 | 0.022 | 0.589 | 20.251 |
| genus.LachnospiraceaeNK4A136group | rs160061 | A | G | 0.051 | 0.011 | 2.12E-06 | -0.014 | 0.022 | 0.53 | 20.246 |
| genus.LachnospiraceaeNK4A136group | rs12611395 | A | G | -0.09 | 0.02 | 5.83E-06 | -0.019 | 0.036 | 0.601 | 19.972 |
| genus.LachnospiraceaeNK4A136group | rs11263806 | A | G | -0.052 | 0.012 | 5.07E-06 | -0.002 | 0.023 | 0.938 | 20.457 |
| genus.LachnospiraceaeNK4A136group | rs10952110 | G | T | 0.049 | 0.011 | 9.08E-06 | 0.023 | 0.022 | 0.302 | 19.758 |
| genus.Lachnoclostridium | rs789029 | C | T | -0.064 | 0.014 | 3.75E-06 | -0.015 | 0.032 | 0.64 | 20.000 |
| genus.Lachnoclostridium | rs78068103 | A | G | 0.089 | 0.019 | 3.67E-06 | 0.006 | 0.034 | 0.874 | 22.630 |
| genus.Lachnoclostridium | rs72829893 | G | T | 0.117 | 0.027 | 5.58E-06 | 0.043 | 0.036 | 0.235 | 19.941 |
| genus.Lachnoclostridium | rs62285313 | A | G | 0.086 | 0.018 | 1.58E-06 | 0.006 | 0.037 | 0.879 | 19.735 |
| genus.Lachnoclostridium | rs61915992 | A | T | 0.08 | 0.017 | 2.67E-06 | 0.052 | 0.031 | 0.098 | 29.336 |
| genus.Lachnoclostridium | rs615997 | T | C | 0.051 | 0.011 | 2.03E-06 | -0.004 | 0.022 | 0.873 | 23.408 |
| genus.Lachnoclostridium | rs6112314 | A | C | -0.056 | 0.011 | 2.43E-07 | -0.012 | 0.023 | 0.604 | 22.809 |
| genus.Lachnoclostridium | rs4738679 | G | A | -0.052 | 0.011 | 4.42E-06 | -0.012 | 0.023 | 0.594 | 21.600 |
| genus.Lachnoclostridium | rs3821998 | C | A | -0.086 | 0.019 | 6.72E-06 | -0.016 | 0.036 | 0.662 | 19.781 |
| genus.Lachnoclostridium | rs2385421 | A | G | 0.075 | 0.018 | 7.14E-06 | -0.043 | 0.034 | 0.205 | 20.036 |
| genus.Lachnoclostridium | rs1997204 | T | C | -0.108 | 0.024 | 5.97E-06 | -0.058 | 0.053 | 0.275 | 20.208 |
| genus.Lachnoclostridium | rs1528479 | G | A | -0.05 | 0.011 | 9.64E-06 | -0.046 | 0.023 | 0.044 | 19.491 |
| genus.Lachnoclostridium | rs12566975 | T | C | -0.047 | 0.011 | 9.57E-06 | -0.003 | 0.022 | 0.885 | 28.367 |
| genus.Lachnoclostridium | rs1031599 | G | T | -0.079 | 0.018 | 6.31E-06 | -0.053 | 0.045 | 0.236 | 20.201 |
| genus.Haemophilus | rs9895850 | T | C | -0.193 | 0.042 | 2.14E-06 | -0.052 | 0.054 | 0.339 | 23.741 |
| genus.Haemophilus | rs9574096 | A | T | -0.074 | 0.016 | 2.18E-06 | -0.012 | 0.023 | 0.605 | 22.630 |
| genus.Haemophilus | rs9382510 | C | T | -0.094 | 0.017 | 7.12E-08 | -0.03 | 0.025 | 0.223 | 17.753 |
| genus.Haemophilus | rs9328464 | T | C | 0.072 | 0.015 | 1.42E-06 | -0.004 | 0.022 | 0.856 | 19.758 |
| genus.Haemophilus | rs78909003 | T | C | -0.246 | 0.05 | 1.67E-06 | 0.013 | 0.049 | 0.783 | 20.745 |
| genus.Haemophilus | rs76022354 | C | T | 0.245 | 0.051 | 1.83E-06 | 0.041 | 0.051 | 0.423 | 19.758 |
| genus.Haemophilus | rs56310940 | G | C | -0.108 | 0.025 | 7.23E-06 | -0.054 | 0.034 | 0.104 | 20.811 |
| genus.Haemophilus | rs4822728 | T | C | 0.071 | 0.015 | 3.48E-06 | 0.032 | 0.022 | 0.147 | 21.788 |
| genus.Haemophilus | rs35509 | G | A | 0.128 | 0.027 | 2.01E-06 | 0.087 | 0.054 | 0.11 | 22.728 |
| genus.Haemophilus | rs12876183 | T | A | 0.075 | 0.017 | 9.62E-06 | 0.044 | 0.023 | 0.058 | 21.425 |
| genus.Haemophilus | rs12191680 | C | G | 0.107 | 0.02 | 1.47E-07 | 0.047 | 0.036 | 0.197 | 23.116 |
| genus.Haemophilus | rs111582866 | G | A | -0.124 | 0.026 | 1.27E-06 | -0.04 | 0.039 | 0.312 | 17.753 |
| genus.Haemophilus | rs10781340 | G | A | 0.095 | 0.02 | 4.32E-06 | -0.05 | 0.033 | 0.137 | 20.531 |
| genus.Erysipelatoclostridium | rs9590927 | G | A | -0.065 | 0.014 | 6.39E-06 | 0.012 | 0.022 | 0.585 | 23.116 |
| genus.Erysipelatoclostridium | rs7221249 | A | G | 0.084 | 0.014 | 4.31E-09 | 0.012 | 0.022 | 0.571 | 19.911 |
| genus.Erysipelatoclostridium | rs710230 | T | C | 0.143 | 0.028 | 6.33E-07 | -0.062 | 0.043 | 0.149 | 22.479 |
| genus.Erysipelatoclostridium | rs6474512 | A | C | 0.067 | 0.014 | 3.02E-06 | -0.02 | 0.023 | 0.383 | 21.896 |
| genus.Erysipelatoclostridium | rs622418 | A | G | -0.067 | 0.014 | 3.68E-06 | 0.043 | 0.022 | 0.052 | 17.685 |
| genus.Erysipelatoclostridium | rs61806970 | C | T | 0.143 | 0.032 | 9.09E-06 | -0.048 | 0.044 | 0.273 | 22.499 |
| genus.Erysipelatoclostridium | rs58236560 | G | T | -0.111 | 0.023 | 2.16E-06 | 0.05 | 0.034 | 0.141 | 20.095 |
| genus.Erysipelatoclostridium | rs4697572 | A | G | -0.081 | 0.016 | 7.59E-07 | -0.015 | 0.028 | 0.589 | 19.578 |
| genus.Erysipelatoclostridium | rs45480394 | T | G | -0.069 | 0.015 | 7.66E-06 | -0.022 | 0.023 | 0.346 | 20.811 |
| genus.Erysipelatoclostridium | rs3804326 | A | G | 0.141 | 0.034 | 9.85E-06 | -0.027 | 0.051 | 0.592 | 19.938 |
| genus.Erysipelatoclostridium | rs34528142 | C | G | -0.088 | 0.02 | 6.13E-06 | 0.044 | 0.027 | 0.107 | 22.594 |
| genus.Erysipelatoclostridium | rs340991 | A | G | -0.074 | 0.016 | 3.75E-06 | 0.059 | 0.025 | 0.017 | 20.743 |
| genus.Erysipelatoclostridium | rs2901723 | C | A | 0.064 | 0.014 | 8.79E-06 | -0.008 | 0.022 | 0.708 | 21.121 |
| genus.Erysipelatoclostridium | rs17804233 | T | C | -0.066 | 0.014 | 4.59E-06 | -0.024 | 0.022 | 0.274 | 20.901 |
| genus.Erysipelatoclostridium | rs16936671 | C | T | -0.097 | 0.022 | 6.04E-06 | 0.042 | 0.032 | 0.19 | 21.266 |
| genus.Erysipelatoclostridium | rs1434153 | G | A | -0.068 | 0.015 | 6.85E-06 | 0.001 | 0.022 | 0.979 | 19.717 |
| genus.Collinsella | rs9541268 | C | A | 0.096 | 0.02 | 8.79E-07 | -0.021 | 0.038 | 0.592 | 20.721 |
| genus.Collinsella | rs75672793 | A | G | -0.109 | 0.024 | 6.14E-06 | 0.035 | 0.052 | 0.503 | 19.940 |
| genus.Collinsella | rs73052258 | G | A | 0.093 | 0.02 | 1.72E-06 | -0.047 | 0.041 | 0.252 | 20.443 |
| genus.Collinsella | rs62448871 | C | A | -0.054 | 0.012 | 6.78E-06 | 0.007 | 0.022 | 0.747 | 20.041 |
| genus.Collinsella | rs59414781 | C | G | 0.067 | 0.015 | 9.15E-06 | -0.054 | 0.032 | 0.093 | 23.867 |
| genus.Collinsella | rs2103510 | G | A | 0.079 | 0.017 | 2.42E-06 | -0.052 | 0.034 | 0.127 | 20.393 |
| genus.Collinsella | rs149807560 | C | A | -0.104 | 0.024 | 7.10E-06 | 0.02 | 0.043 | 0.651 | 20.464 |
| genus.Collinsella | rs1496626 | T | C | -0.072 | 0.016 | 6.78E-06 | -0.021 | 0.032 | 0.523 | 24.786 |
| genus.Collinsella | rs12921100 | A | T | 0.056 | 0.013 | 8.23E-06 | -0.016 | 0.025 | 0.53 | 19.715 |
| genus.Collinsella | rs11597285 | G | T | -0.054 | 0.012 | 9.38E-06 | 0.01 | 0.022 | 0.663 | 21.245 |
| genus.Collinsella | rs10890671 | T | C | -0.054 | 0.012 | 6.52E-06 | 0.022 | 0.022 | 0.325 | 19.940 |
| family.Erysipelotrichaceae | rs8003149 | C | T | 0.054 | 0.012 | 4.08E-06 | 0.022 | 0.023 | 0.34 | 20.525 |
| family.Erysipelotrichaceae | rs7826267 | T | G | -0.084 | 0.02 | 9.28E-06 | -0.021 | 0.045 | 0.635 | 24.091 |
| family.Erysipelotrichaceae | rs7234058 | T | C | -0.095 | 0.019 | 9.12E-07 | -0.059 | 0.038 | 0.125 | 19.542 |
| family.Erysipelotrichaceae | rs62504403 | C | T | 0.068 | 0.013 | 1.12E-07 | -0.013 | 0.028 | 0.64 | 19.751 |
| family.Erysipelotrichaceae | rs56970041 | T | G | 0.072 | 0.016 | 5.40E-06 | -0.027 | 0.046 | 0.559 | 22.652 |
| family.Erysipelotrichaceae | rs4078432 | C | T | -0.061 | 0.013 | 4.23E-06 | 0.005 | 0.029 | 0.866 | 17.044 |
| family.Erysipelotrichaceae | rs35161940 | T | C | -0.081 | 0.017 | 1.85E-06 | -0.023 | 0.036 | 0.521 | 21.897 |
| family.Erysipelotrichaceae | rs290833 | T | G | -0.05 | 0.011 | 8.03E-06 | 0.007 | 0.022 | 0.757 | 19.813 |
| family.Erysipelotrichaceae | rs2300774 | G | A | 0.052 | 0.011 | 8.95E-07 | 0.007 | 0.022 | 0.736 | 20.541 |
| family.Erysipelotrichaceae | rs1884466 | C | T | -0.048 | 0.011 | 9.53E-06 | -0.03 | 0.022 | 0.171 | 21.569 |
| family.Erysipelotrichaceae | rs17530232 | A | G | 0.103 | 0.022 | 2.79E-06 | 0.002 | 0.05 | 0.963 | 20.947 |
| family.Erysipelotrichaceae | rs10781552 | C | T | -0.055 | 0.012 | 2.33E-06 | -0.076 | 0.024 | 0.002 | 22.073 |
| family.Erysipelotrichaceae | rs1074800 | A | G | 0.049 | 0.011 | 6.15E-06 | 0.004 | 0.022 | 0.846 | 22.542 |
| class.Gammaproteobacteria | rs9973122 | T | A | 0.074 | 0.016 | 6.77E-06 | -0.003 | 0.04 | 0.949 | 28.367 |
| class.Gammaproteobacteria | rs9494710 | C | T | -0.055 | 0.012 | 4.55E-06 | 0.037 | 0.024 | 0.116 | 19.383 |
| class.Gammaproteobacteria | rs79795896 | A | G | -0.159 | 0.035 | 7.92E-06 | 0.091 | 0.052 | 0.08 | 21.658 |
| class.Gammaproteobacteria | rs75101789 | C | T | 0.073 | 0.016 | 8.79E-06 | 0.027 | 0.039 | 0.488 | 21.064 |
| class.Gammaproteobacteria | rs6706173 | A | C | 0.074 | 0.015 | 1.99E-07 | -0.055 | 0.032 | 0.086 | 20.159 |
| class.Gammaproteobacteria | rs6469506 | T | A | 0.054 | 0.012 | 3.29E-06 | -0.02 | 0.023 | 0.384 | 20.268 |
| class.Gammaproteobacteria | rs12404135 | A | G | -0.079 | 0.017 | 8.89E-06 | 0.023 | 0.041 | 0.573 | 24.091 |
| class.Gammaproteobacteria | rs11181912 | G | A | -0.058 | 0.012 | 9.95E-07 | -0.001 | 0.023 | 0.964 | 19.383 |
| class.Erysipelotrichia | rs8003149 | C | T | 0.054 | 0.012 | 4.08E-06 | 0.022 | 0.023 | 0.34 | 25.723 |
| class.Erysipelotrichia | rs7826267 | T | G | -0.084 | 0.02 | 9.28E-06 | -0.021 | 0.045 | 0.635 | 23.741 |
| class.Erysipelotrichia | rs7234058 | T | C | -0.095 | 0.019 | 9.12E-07 | -0.059 | 0.038 | 0.125 | 20.161 |
| class.Erysipelotrichia | rs62504403 | C | T | 0.068 | 0.013 | 1.12E-07 | -0.013 | 0.028 | 0.64 | 19.754 |
| class.Erysipelotrichia | rs56970041 | T | G | 0.072 | 0.016 | 5.40E-06 | -0.027 | 0.046 | 0.559 | 19.246 |
| class.Erysipelotrichia | rs4078432 | C | T | -0.061 | 0.013 | 4.23E-06 | 0.005 | 0.029 | 0.866 | 19.195 |
| class.Erysipelotrichia | rs35161940 | T | C | -0.081 | 0.017 | 1.85E-06 | -0.023 | 0.036 | 0.521 | 21.118 |
| class.Erysipelotrichia | rs290833 | T | G | -0.05 | 0.011 | 8.03E-06 | 0.007 | 0.022 | 0.757 | 22.644 |
| class.Erysipelotrichia | rs2300774 | G | A | 0.052 | 0.011 | 8.95E-07 | 0.007 | 0.022 | 0.736 | 23.255 |
| class.Erysipelotrichia | rs1884466 | C | T | -0.048 | 0.011 | 9.53E-06 | -0.03 | 0.022 | 0.171 | 20.501 |
| class.Erysipelotrichia | rs17530232 | A | G | 0.103 | 0.022 | 2.79E-06 | 0.002 | 0.05 | 0.963 | 20.366 |
| class.Erysipelotrichia | rs10781552 | C | T | -0.055 | 0.012 | 2.33E-06 | -0.076 | 0.024 | 0.002 | 27.358 |
| class.Erysipelotrichia | rs1074800 | A | G | 0.049 | 0.011 | 6.15E-06 | 0.004 | 0.022 | 0.846 | 21.559 |
